# Clinical efficacy of plasma exchange in systemic lupus erythematosus during pregnancy

**DOI:** 10.1002/iid3.1041

**Published:** 2023-10-11

**Authors:** Fen Zhang, Bing‐Ying Zhang, Ru Fan, Ting Cheng, Xiao‐Rong Hu, Yu‐Qing Liu, Xing Cen, Yu‐Jie Bu, Jian‐Ping Cao, Feng‐Wu Chen, Jun‐Wei Chen

**Affiliations:** ^1^ Department of Rheumatology The Second Hospital of Shanxi Medical University Taiyuan City Shanxi Province China; ^2^ Department of Respiratory Medicine Xinzhou People's Hospital Xinzhou City Shanxi Province China; ^3^ State Key Laboratory of Multiphase Flow in Power Engineering Xi'an Jiaotong University Xi'an Shanxi Province China

**Keywords:** efficacy, plasmapheresis, pregnancy, systemic lupus erythematosus

## Abstract

**Objective:**

To investigate the clinical efficacy of plasma exchange (PE) with or without prednisone and hydroxychloroquine (HCQ) for the treatment of systemic lupus erythematosus (SLE) during pregnancy.

**Methods:**

The clinical characteristics of 14 pregnant women with SLE admitted to our hospital were retrospectively analyzed, including 7 only treated with prednisone and HCQ (non‐PE group) as well as 7 combined PE (PE group). The delivery situations of 14 patients were recorded. Data like erythrocyte sedimentation rate (ESR), urine protein, platelet count, and SLEDAI scores were compared between two groups before treatment and 3, 6, and 12 months after delivery.

**Results:**

Three patients in the non‐PE group ended in miscarriage while all patients in the PE group were delivered successfully. Eleven successfully delivered fetuses in the two groups were healthy, and the Apgar scores were over 8. The ESR of the PE group was significantly lower than that of the non‐PE group at 6 and 12 months after delivery, while there was no statistical difference in ESR between the two groups before treatment and 3 months after delivery. The ESR and urine protein were significantly higher in the non‐PE group at months 3, 6, and 12 postpartum. There was a significant decrease in disease activity postpartum in the PE group compared to predelivery disease activity. The change in platelet counts between the two groups significantly increased over time in the PE group, while SLEDAI scores decreased.

**Conclusions:**

The combination of PE and oral prednisone and HCQ is possibly a more effective treatment than oral prednisone and HCQ alone for patients with active SLE during pregnancy. This treatment option reduces pregnancy loss and promotes the patients' postpartum condition to a certain extent.

## Key messages

Systemic lupus erythematosus (SLE) is a complicated autoimmune disease, that leads to an increased probability of pregnancy loss and adverse pregnancy outcomes. The use of a small number of immunosuppressants and biological agents during pregnancy probably puts the fetuses' of SLE at risk for teratogenesis. This dramatically limits drug treatment during pregnancy. Plasmapheresis (PE) can effectively remove pathogenic substances in plasma with high molecular weights, such as autoantibodies, immune complexes, and cryoglobulin. In our study, the combination of PE and oral prednisone and HCQ may be a more effective treatment than oral prednisone and HCQ alone for patients with active SLE during pregnancy. To a certain extent, this treatment option reduces pregnancy loss and promotes the patients' postpartum condition.

## Graphical abstract

In this study, we recruit 14 SLE patients to analyze the clinical efficacy of prednisone and HCQ alone or in combination with PE for the treatment of SLE during pregnancy. The data showed that PE in the combination of PE and oral prednisone and HCQ is a more effective treatment than oral prednisone and HCQ alone for patients with active SLE during pregnancy. This treatment option reduces pregnancy loss and promotes the patients' postpartum condition.

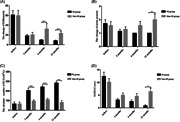

## 1 INTRODUCTION

Systemic lupus erythematosus (SLE) is a complicated autoimmune disease characterized by systemic multiple‐organ damage, which can easily occur in women of childbearing age. SLE before and during conception and pregnancy may aggravate the condition, leading to adverse pregnancy outcomes and an increased probability of pregnancy loss.^1^ It has been reported that miscarriage, fetal mortality, and preterm birth rate of SLE patients are higher than the average person, reaching 20%～30%.^2^, ^3^, ^4^ Further, the use of a small number of immunosuppressants and biological agents during pregnancy probably puts the fetuses' of SLE at risk for teratogenesis.^5^, ^6^ This greatly limits drug treatment during pregnancy. Plasmapheresis (PE) can effectively remove pathogenic substances in plasma with high molecular weights, such as autoantibodies, immune complexes, and cryoglobulin. There have been successful cases of PE improving the disease activity and pregnancy outcome of SLE patients.^7^, ^8^ However, most of them were case reports. In our study, we analyzed the successful administration of a treatment regimen involving PE to seven pregnant SLE patients in our hospital and compared treatment efficacy to a control group who did not receive PE. We propose that this regimen may be a safe and effective method to promote successful pregnancy outcomes in SLE patients.

## 2 MATERIALS AND METHODS

### 2.1 Participants

Fourteen pregnant SLE patients were treated in the Second Hospital of Shanxi Medical University from October 2017 to January 2020. The disease activity of SLE patients was assessed using the SLE Disease Activity Index 2000 update (SLEDAI‐2K) score. This study has been approved by the Ethics Committee of the Second Hospital of Shanxi Medical University and obtained the informed consent of all participants, serial number 2023YX111. The relevant information on the 14 pregnant SLE patients was obtained based on their hospitalization history and outpatient medical records (Table 1). The laboratory tests, treatment, outcomes, and delivery of all patients were retrospectively analyzed. The 14 patients were divided into 2 groups, the PE and non‐PE groups.

**Table 1:** Essential features before treatment of 14 SLE patients with pregnancy.

		Age (years)	Positive antibodies	diagnosis	Onset time/delivery time
PE group	1	27	ANA1:1280HS, anti‐dsDNA antibody, ACA, anti‐ß2GPI antibody, P‐ANCA	SLE, ST	25^+^/36^+2^ weeks
	2	20	ANA1:320HS, anti‐ß2GPI antibody, P‐ANCA, anti ‐SSA, Ro52	SLE, LN	22^+^/33^+2^ weeks
	3	24	ANA1:1280H, anti‐dsDNA antibody, ACA, anti‐ß2GPI antibody, anti‐SSA, anti‐SSA antibody	SLE, LN	25^+1^/38^+^ weeks
	4	30	ANA1:160S, ACA, anti‐SSA, Ro52	SLE, Pericardial effusion	3/38^+1^ weeks
	5	26	ANA > 1:1280H, anti‐dsDNA antibody, ACA, anti‐ß2GPI antibody, P‐ANCA,	SLE, ST	38^+1^/38^+6^ weeks
	6	32	ANA > 1:1280S, anti‐SSA, anti‐SSB, anti‐Ro52	SLE, ST	31^+2^/37^+2^ weeks
	7	25	ANA1:1280S, anti‐dsDNA antibody, anti‐ß2GPI antibody	SLE, LN, portal vasculitis	22/40^+3^ weeks
Non‐PE group	1	32	ANA1:160HS, anti‐dsDNA antibody, ACA, anti‐SSA, anti‐Ro52, α‐CCP	SLE	36/39^+2^ weeks
	2	32	ANA1:1280S, anti‐SSA/Ro50KD antibody, anti‐SSA/Ro60KD antibody, anti‐UI‐snRNP antibody	SLE	24/27^+3^ weeks
	3	28	ANA1:1280S, anti‐dsDNA antibody, anti‐SSA, anti‐SSB, anti‐Ro52 antibody	SLE, LN	35/37^+1^ weeks
	4	26	ANA1:640H, anti‐SSA antibody, anti‐SSA	SLE, LN	25/34 weeks
	5	24	ANA1:640S, anti‐dsDNA antibody, anti‐SSA antibody, anti‐SSA, anti‐RNP, anti‐U1RNP	SLE	20/37 weeks
	6	23	ANA1:1280S, anti‐dsDNA antibody	SLE, ST	5/7^+2^ weeks
	7	31	ANA1:1280S, anti‐dsDNA antibody, P‐ANCA	SLE, LN, severe pre‐eclampsia	27/28^+4^ weeks

Abbreviations: ACA, anticardiolipin antibody; ANA, antinuclear antibody; LN, lupus nephritis; SLEDAI, systemic lupus erythematosus disease activity index; ST, secondary thrombocytopenia.

### 2.2 Inclusion and exclusion criteria

Inclusion criteria: (1) All patients conformed to the American College of Rheumatology 1997 revised criteria for the classification of SLE. (2) All patients had no significant organ involvement before pregnancy and maintained stable conditions for 6 months or more. They had taken oral prednisone dose of less than 10 mg and had stopped taking immunosuppressive agents (such as cyclophosphamide, methotrexate, mycophenolate mofetil, etc.) for more than 6 months before conception. It was also ensured that their renal function was stable (serum creatinine level was normal or glomerular filtration rate was higher than 60 mL min^−1^, and 24‐h urine protein was less than 0.5 g/d). (3) They developed disease activity during pregnancy.

Exclusion criteria: (1) Those using immunosuppressants and drugs other than glucocorticoids and hydroxychloroquine. (2) Presented with other autoimmune diseases (such as rheumatoid arthritis), tuberculosis, drug‐induced lupus, and malignant tumors. (3) Presented with other diseases related to abortion, such as chromosomal abnormalities, endocrine disorders, and uterine malformations. (4) Allergic to the treatment drugs in this study.

### 2.3 Experimental method

#### 2.3.1 Collecting clinical data

The patients in the PE group underwent oral prednisone (5–60 mg/day), HCQ (200 mg, twice a day) and PE (once every other day) treatments, while the patients in the non‐PE group received oral prednisone (5–60 mg/day) and HCQ (200 mg, twice a day) when they presented with disease activity before childbirth. The method of PE is double‐filtration plasmapheresis (DFPP) (two times) and immunoadsorption (IA) (one time). Postpartum, PE was stopped in all patients, and the dose of prednisone was adjusted according to the patients' condition in both groups. Simultaneously, information on clinical symptoms was collected in detail and SLEDAI scores were calculated. ESR, urine protein, and platelet counts were analyzed 3, 6, and 12 months postpartum to evaluate the differences between the two groups.

#### 2.3.2 PE method

**Figure 1:**
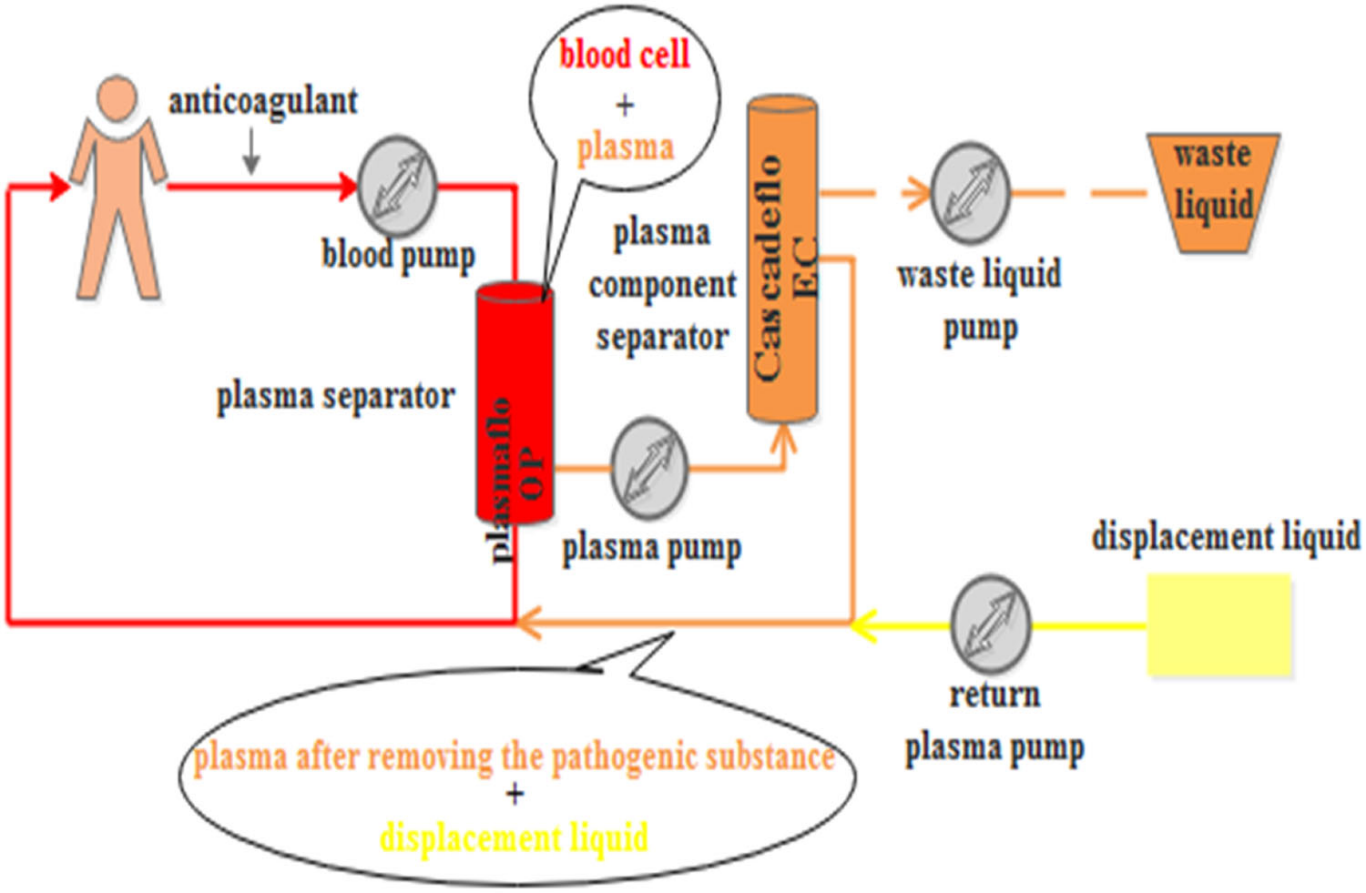
The schematic of DFPP. DFPP, double‐filtration plasmapheresis.

**Figure 2:**
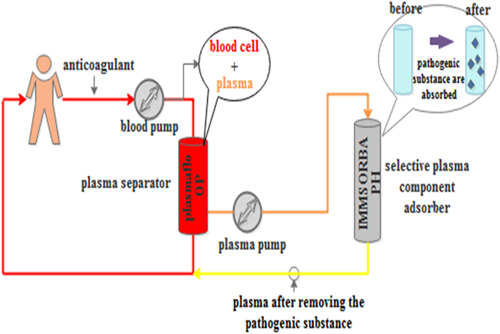
The schematic of the IA. IA, immunoadsorption.

PlasautoΣ from Asahi Kasei Corporation of Japan were the DFPP and IA blood purification devices used. The Plasmaflo OP from Asahi Kasei was used as the first‐stage plasma separator for DFPP and IA. The DFPP secondary plasma component separator was Cascadeflo EC‐30 type from Asahi Kasei. The blood flow in the secondary plasma separator was maintained at 30 mL/min, and the amount of pulp was 500 mL each time, using 5% albumin in saline as a replacement (Figure 1). The IA plasma component adsorber used was Immusorba PH from Asahi Kasei Corporation (Figure 2).

In this study, plasma exchange was performed every other day in the PE group, and the plasma volume of each patient was 2 L each time, and the time of plasma treatment was 2 h each time. The total sessions should be determined by the patients' clinical symptoms, laboratory indexes, and disease remission. All the patients in our experiment underwent two times of DFPPs and one time of IA.

### 2.4 Statistical analysis

Data analysis was performed using SPSS 24.0 statistical software. Quantitative data conforming to the normal distribution was represented as mean ± standard deviations (SD). The comparison within the group was performed by repeated measures ANOVA. The comparisons between groups were analyzed by one‐way ANOVA. A pairwise comparison was performed using the least—significant difference (LSD) method. Non‐normally distributed data were represented as M (Q25, Q75), and the comparison between multiple groups was performed by the Kruskal–Wallis *H* test. *p* < .05 was considered significant.

## 3 RESULTS

There were seven pregnant females in the PE group with mean age [26.0 (24.0,30.0) years], ranging from 20 to 32 years, and mean disease course [48.0 (15,102.0) months], ranging from 14 to 156 months. Similarly, there were seven pregnant females in the non‐PE group, mean age [28.0 (24.0,32.0) years], ranging from 23 to 32 years, and suggested disease course [60.0 (36.0,120.0) months], ranging from 36 to 120 months. The two groups had no significant differences in age (*p* = .45), disease course (*p* = .55), and childbearing history.

### 3.1 Childbirth and disease activity in mothers postpartum

All patients in the PE group delivered successfully. However, three of the seven patients in the non‐PE group ended in miscarriage, and the remaining four delivered successfully. The 11 fetuses in the study developed well and were born with an APGAR score of 8 or more at birth. PE group recovered well and the disease was in remission within 1 year after delivery. Conversely, the four patients in the non‐PE group developed disease recurrence within 1‐year postpartum.

### 3.2 Efficacy of PE in the treatment of patients with SLE during pregnancy

**Figure 3:**
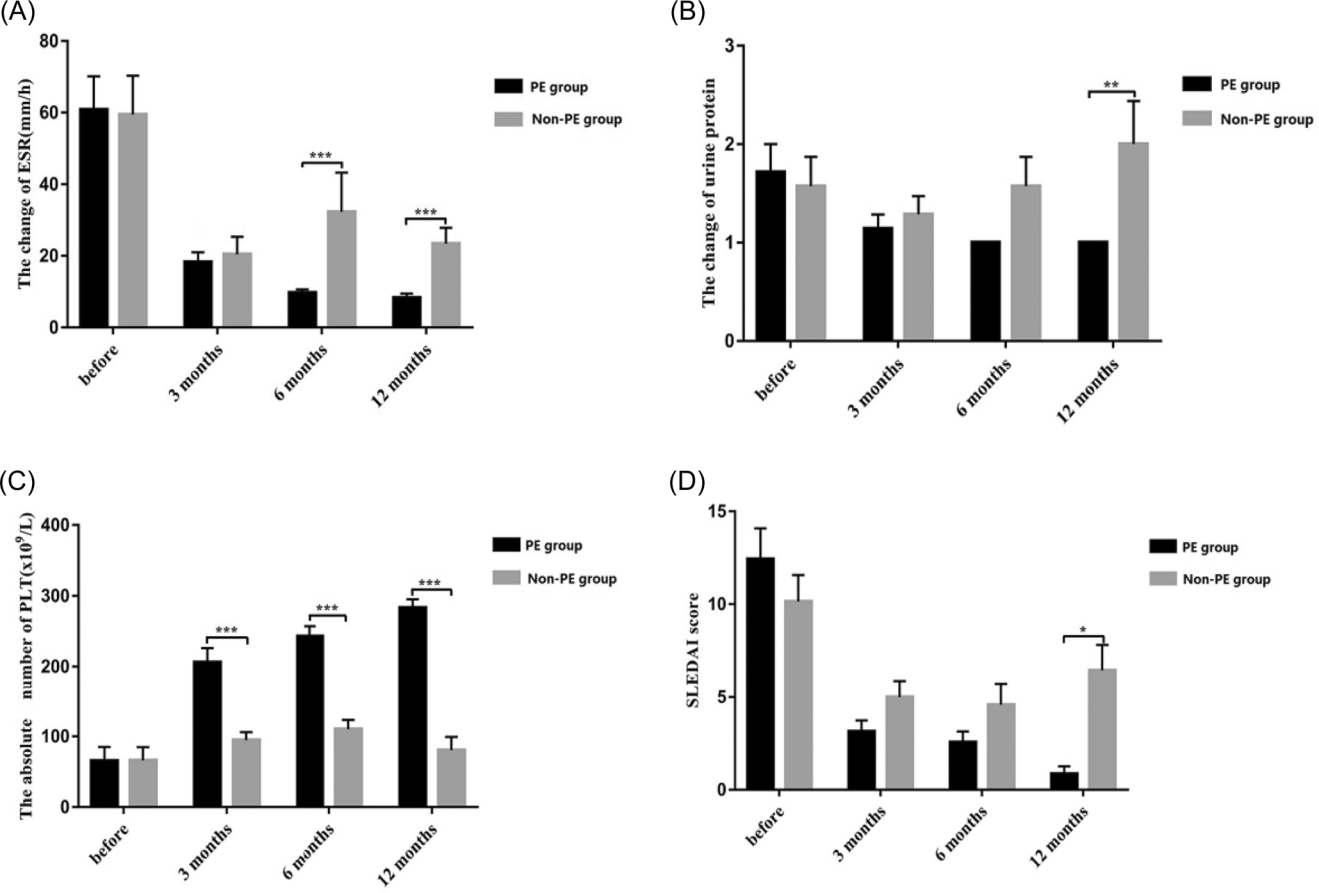
Clinical characteristics before treatment as well as 3, 6, and 12 months after delivery in both groups. (A) Changes in ESR; (B) urine protein changes; (C) changes in platelet count; (D) changes in SLEDAI scores. ESR, erythrocyte sedimentation rate.

To assess the efficacy of the two treatment regimens, we analyzed the changes in ESR, urine protein, platelet count, and SLEDAI scores before treatment as well as 3, 6, and 12 months after delivery in both groups (Table 2). As is shown in Figure 3A, the changes of ESR between the two groups were not statistically significant in the main effect of grouping (*p* = .114) and the interaction effect between grouping and time (*p* = .245). However, there was a statistical difference in different time points (*p* < .001). In other words, there were statistical differences between the two groups at 3, 6, and 12 months after delivery, and ESR in the non‐PE group was higher than in the PE group. Compared to the ESR before treatment (60.86 ± 24.63), the ESR at month 3 (18.29 ± 7.16), 6 (9.71 ± 2.56), and 12 (8.29 ± 3.30) postpartum had significantly declined in the PE group, showing a gradual downward trend. However, in the non‐PE group, there was a significant difference only at month 3 (20.43 ± 13.15) and 12 (23.43 ± 11.69) after delivery when compared to the ESR before delivery (59.43 ± 28.87). There was no further decline at months 6 and 12 after delivery compared to month 3 in the non‐PE group.

**Table 2:** Clinical characteristics before treatment as well as 3, 6, and 12 months after delivery of 14 SLE patients with pregnancy.

			ESR (mm/h)				Urine protein				SLEDAI score				Platelet count		
		BT	AD‐3	AD‐6	AD‐12	BT	AD‐3	AD‐6	AD‐12	BT	AD‐3	AD‐6	AD‐12	BT	AD‐3	AD‐6	AD‐12
PE group	1	30	7	11	9	‐	‐	‐	‐	10	2	2	0	28	140	267	308
	2	58	20	6	5	‐	‐	‐	‐	12	4	4	2	120	215	280	301
	3	88	11	10	7	+	‐	‐	‐	19	2	0	0	90	177	198	298
	4	32	17	9	6	‐	‐	‐	‐	8	2	2	0	102	308	277	297
	5	53	27	12	7	+	‐	‐	‐	9	4	4	2	4	208	189	215
	6	75	22	13	9	+	‐	‐	‐	11	2	2	0	3	218	256	278
	7	90	24	7	15	++	+	‐	‐	18	6	4	2	110	178	234	286
Non‐PE group	1	72	18	15	20	‐	‐	‐	+	6	4	4	8	20	79	128	84
	2	108	21	25	14	‐	‐	‐	‐	6	2	0	2	15	68	80	46
	3	53	43	32	41	+	+	++	+	16	8	4	8	7	62	140	88
	4	43	32	26	30	+	+	+	++	12	8	6	8	89	96	110	68
	5	80	5	96	28	‐	‐	‐	‐	10	4	8	12	96	88	67	52
	6	27	10	11	5	‐	‐	‐	‐	8	5	2	5	112	140	87	38
	7	33	14	21	26	++	‐	+	+++	13	4	8	2	122	129	160	186

Abbreviations: AD‐3, 3 months after delivery; AD‐6, 6 months after delivery; AD‐12, 12 months after delivery; BT, before treatment.

As is shown in Figure 3B, the urine protein concentration in the two groups were not statistically significant in the main effect of grouping (*p* = .186) and time (*p* = .101). The interaction effect of grouping and time was statistically significant (*p* = .027). Further analysis showed that the two groups were statistically significant at month 12 after delivery, and the values of the non‐PE group were higher than the PE group. Compared to the urine protein before delivery, there were statistical differences at months 3, 6, and 12 postpartum in the PE group, which gradually decreased. However, there were no statistical differences in the non‐PE group.

As is depicted in Figure 3C, the platelet counts between the two groups were statistically significant in the main effect of grouping and time and in the interaction effect between grouping and time (*p* < .001). Further analysis of the interaction showed that the platelet counts of the two groups were statistically significant at months 3, 6, and 12 postpartum compared to the counts before delivery. There were statistical differences at months 3 (206.29 ± 52.62), 6 (243 ± 37.18), and 12 (283.29 ± 31.70) postpartum when compared to the counts before delivery (65.29 ± 51.60) in the PE group, showing a gradually increasing trend. In the non‐PE group, there was a statistically significant decrease at month 12 (80.29 ± 50.27) postpartum compared to month 6 (110.29 ± 34.16) postpartum.

As is shown in Figure 3D, The SLEDAI scores of the two groups were calculated. The two groups were not statistically significant in the main effect of grouping (*p* = .119) and the interaction effect of grouping and time (*p* = .060). There was, however a statistically significant difference at different time points (*p* < .001). The SLEDAI scores of the non‐PE group, especially postpartum, were higher than the PE group. Compared to the scores before delivery (12.43 ± 4.35), the SLEDAI score at month 3 (3.14 ± 1.57), 6(2.57 ± 1.51), and 12 (0.86 ± 1.07) after delivery declined significantly in the PE group. In the non‐PE group, the SLEDAI score at month 3 (5.00 ± 2.24) and 6 (4.57 ± 2.99) postpartum were lower than the score before delivery (10.14 ± 3.76), however, they increased at month 12 (6.43 ± 3.65) compared to month 6 postpartum.

## 4 DISCUSSION

SLE is a systemic autoimmune disease which easily occurs in women of childbearing age and is associated with systemic damage. Although its etiology is very complex and still unclear, the primary pathogenesis is the production of many autoantibodies and the deposition of immune complexes. Related studies believed that the production of many autoantibodies in SLE patients would not affect their pregnancy ability, but they were highly prone to relapse during pregnancy.^9^ A study of 113 pregnant women with previous lupus nephritis (LN) found that 30% of patients worsened during or after pregnancy. In contrast, other studies showed that the possibility of deterioration was as high as 60%.^9^ Second, an increase in adverse pregnancy outcomes and maternal and child complications can be expected if SLE patients demonstrate disease activity during pregnancy.^10^ Therefore, it is critical to develop a rapid and effective treatment plan when SLE patients have disease recurrence during pregnancy. However, drugs known as standard treatment (standard of care [SoC]) of SLE, such as hormones, antimalarial drugs and immunosuppressants, not only have shortcomings such as large side effects, poor targeting, high disease recurrence rate, and so on,^11^ but also have certain restrictions or even are contraindicated for pregnant women. So, it is very crucial to explore a rapid, effective, and safe treatment for SLE patients and fetuses.

Studies have confirmed that PE is a rapid and effective method for the treatment of SLE.^12^, ^13^ PE includes simple plasma exchange, DFPP, and IA, which can remove various pathological components of blood circulation in a short time. Simple plasma exchange requires abundant plasma preparations in the course of treatment, which has a high risk of infection and allergy, so generally it is not used in pregnant women. DFPP can selectively remove plasma macromolecular substances, such as IgG, various antibodies, and immune complexes, and return filtered albumin and small molecular substances back to patients. It can also effectively improve the clinical symptoms of SLE patients. Immusorba PH350 uses phenylalanine as a ligand and binds to immunoglobulin through hydrophobic affinity. It has high selectivity to immune complex, anticardiolipin antibodies and anti‐SSA antibodies. Previous studies have shown that plasmapheresis could be achieved by scavenging SSA or SSB antibodies to prevent CHB^14^ in the fetus of SLE patients with pregnancy. Moreover, there have also been reports that IA can successfully treat recurrent pregnancy loss by removing anticardiolipin antibodies.^15^ In this paper, the combination of DFPP and IA in the treatment of SLE with pregnancy can remove a variety of immune complexes, autoantibodies and other pathogenic substances, which not only makes up for the slow efficacy of standard treatment (oral low‐dose hormone, HCQ), but also avoids the side effects and adverse pregnancy outcomes caused by the use of immunosuppressants. Based on the above theoretical support, this study analyzed the efficacy of DFPP and IA in the treatment of SLE during pregnancy. The results showed that seven patients in the PE group treated with DFPP and IA were delivered successfully, and the fetus was in good health with no complications, while three of the seven patients in the non‐PE group treated with oral low‐dose hormone and HCQ had an abortion during pregnancy, which indicated the necessity of DFPP and IA therapy in the treatment of SLE with pregnancy. It can reduce the occurrence of adverse pregnancy outcomes. Regarding the safety of maternal and infant, the results of a study^16^ affirmed that the application of therapeutic apheresis (including PE) during pregnancy is safe for mothers and fetuses. In this study, seven patients in the PE group had no related complications in the course of treatment and achieved good results.

In this study, compared with the non‐PE group, the levels of ESR and urinary protein in the PE group were lower than those in the non‐PE group at 3, 6, and 12 months after delivery, and the platelet count was higher than that in the non‐PE group at 3, 6, and 12 months after delivery. After consulting the literature, two cases reported that the results were similar to those of this study. Among them, one SLE patient^7^ with a previous history of thrombotic thrombocytopenic purpura (TTP) received prophylactic PE treatment eight times a month since the eighth week of pregna. No disease activity occurred during pregnancy, and the platelet count remained at the normal level. In another case,^8^ LN recurred during pregnancy, with abnormal renal function, positive urinary protein, and gestational hypertension. Still, the condition was controlled, the urine protein and renal function were improved, and the delivery was successful after PE treatment. The therapeutic effects of the two groups were evaluated comprehensively by calculating the SLEDAI score in this study. It was found that compared with the non‐PE group, the SLEDAI score of the PE group was lower than that in the non‐PE group at 3, 6, and 12 months after delivery. The SLEDAI score of the non‐PE group at 12 month was higher than that at 6 month after delivery, which indicated that DFPP combined with IA therapy could reduce the disease activity of SLE patients with pregnancy. The use of oral drugs alone may lead to an increase in disease activity. A follow‐up study^17^ of 237 SLE patients treated with prednisone, HCQ, and azathioprine during pregnancy found that SLE disease activity increased at 6‐ and 12‐month postpartum compared to antenatal and immediately after delivery. Therefore, DFPP and IA may be an effective method for the treatment of SLE with pregnancy, which can not only improve clinical indexes such as ESR, urinary protein, and platelet count but also keep the disease stable for a long time. DFPP and IA may play a role in the following ways: (1) to remove a variety of autoantibodies and immune complexes, (2) to remove cytokines and other inflammatory factors in the body (3) to regulate the immune function of patients.

In addition, antiphospholipid antibodies were positive in six patients in the PE group, while only one patient in the other group was positive. For SLE patients, secondary antiphospholipid syndrome (APS) should not only be considered positive for laboratory antibodies, but also be consistent with clinical symptoms such as repeated intravascular thrombosis or habitual abortion. None of the patients in our study were complicated with secondary APS, we think that PE probably removes antiphospholipid antibodies and prevents secondary APS complications, such as abortion and placental dysfunction.

While this study has achieved some research results, there are also some shortcomings. First, the number of samples in this study is small, if we can increase the sample size, it may be of more clinical significance. Second, the analytical time of this study is relatively short. Therefore, we will continue to collect such cases in the next work and analyze the changes of SLE patients' condition in longer postpartum period.

In conclusion, combined with research data and clinical experience, DFPP combined with IA maybe a valuable treatment for patients with SLE during pregnancy.

## AUTHOR CONTRIBUTIONS

All authors contributed to the article, and authors (Fen Zhang, Bing‐Ying Zhang, Ru Fan, Ting Cheng, Xiao‐Rong Hu, Yu‐Qing Liu, Xing Cen, Yu‐Jie Bu, Jian‐Ping Cao, Feng‐Wu Chen, Jun‐Wei Chen) have read and approved the submitted version. Study design and manuscript writing: Fen Zhang, Bing‐Ying Zhang, and Ting Cheng. Data extraction, quality assessment, analysis, and interpretation of data: Ru Fan, Xiao‐Rong Hu, Yu‐Qing Liu, Jian‐Ping Cao, Xing Cen, Yu‐Jie Bu, Feng‐Wu Chen. Mechanism of PE and formulation of PE treatment plan: Jian‐Ping Cao and Feng‐Wu Chen. All authors were involved in drafting the article or revising the important intellectual content critically and all authors approved the final version to be published. Jun‐Wei Chen takes responsibility for the integrity of the data and the accuracy of the analysis.

## CONFLICT OF INTEREST STATEMENT

The authors declare no conflict of interest.

## ETHICS STATEMENT

Ethics Committee of the second Hospital of Shanxi Medical University; reference number: 2023YX111.

## Supporting information

Supporting information.
