# Supplementary material for: Clinical efficacy of plasma exchange in systemic lupus erythematosus during pregnancy
Source: Immun Inflamm Dis. 2023 Oct 11;11(10):e1041. doi: 10.1002/iid3.1041 (PMC10566447; doi:10.1002/iid3.1041)
Supplement: Supplementary file 1 — Supporting information. [file IID3-11-e1041-s001.docx]

**TableS1. Childbearing history and the delivery situation of 14 SLE patients**

|  | | Childbearing history | Delivery situation | | | | Pregnancy complications | | |
| --- | --- | --- | --- | --- | --- | --- | --- | --- | --- |
|  |  |  | Delivery mode | Apgar score | Neonatal weight (kg) | Neonatal condition | Gestational hypertension | Preeclampsia | Gestational diabetes mellitus |
| PE group | 1 | G_1_P_0_ | C-sect | 9 | 2.46 | Sound | None | None | None |
|  | 2 | G_2_P_0_ abortion 1 | C-sect | 9 | 2.32 | Sound | None | Yes | None |
|  | 3 | G_1_P_0_ | C-sect | 10 | 1.98 | Sound | None | None | None |
|  | 4 | G_2_P_0_ abortion 1 | C-sect | 8 | 3.02 | Sound | Yes | None | None |
|  | 5 | G_2_P_0_ abortion 1 | C-sect | 9 | 3.23 | Sound | None | None | None |
|  | 6 | G_1_P_1_ | C-sect | 9 | 1.68 | Sound | Yes | None | None |
|  | 7 | G_1_P_0_ | C-sect | 10 | 3.69 | Sound | None | None | None |
| Non-PE group | 1 | G_3_P_1_ abortion 1 | C-sect | 9 | 2.65 | Sound | Yes | None | None |
|  | 2 | G_2_P_1_ | Abortion | — | — | — | None | None | Yes |
|  | 3 | G_1_P_0_ | C-sect | 8 | 3.12 | Sound | None | None | None |
|  | 4 | G_1_P_0_ | C-sect | 9 | 2.71 | Sound | None | None | None |
|  | 5 | G_2_P_0_ abortion 1 | C-sect | 9 | 2.12 | Sound | None | None | None |
|  | 6 | G_1_P_0_ | Abortion | — | — | — | None | None | None |
|  | 7 | G_2_P_0_ abortion 1 | Abortion | — | — | — | None | None | Yes |
| C-sect: cesarean section, Apgar score: 8-10 points are normal, 4-7 are mild asphyxia, 0-3 are severe asphyxia, -: does not exist. | | | | | | | |  |  |

**Table S2. Detailed therapy description of 14 SLE patients with pregnancy**

| **Group** | **SN** | **AD** | **Therapy during hospitalization** | **Therapy of 3 months after delivery** | **Therapy of 6 months after delivery** | **Therapy of 12 months after delivery** |
| --- | --- | --- | --- | --- | --- | --- |
| **PE group** | 1 | 772933 | DFPP (2 times) and IA (1 time);  Prednisone (60 mg, po, once a day); HCQ (200 mg, po, twice a day) | Prednisone (40 mg, po, once a day); HCQ (200 mg, po, twice a day) | Prednisone (30 mg, po, once a day); HCQ (200 mg, po, twice a day) | Prednisone (20 mg, po, once a day); HCQ (200 mg, po, twice a day) |
|  | 2 | 784832 | DFPP (2 times) and IA (1 time); Prednisone (60 mg, po, once a day); HCQ (200 mg, po, twice a day) | Prednisone (20 mg, po, once a day); HCQ (200 mg, po, twice a day) | Prednisone (10 mg, po, once a day); HCQ (200 mg, po, twice a day) | Prednisone (10 mg, po, once a day); HCQ (200 mg, po, twice a day) |
|  | 3 | 721834 | DFPP (2 times) and IA (1 time); Prednisone (20 mg, po, once a day); HCQ (200 mg, po, twice a day) | Prednisone (10 mg, po, once a day); HCQ (200 mg, po, twice a day) | Prednisone (5 mg, po, once a day); HCQ (200 mg, po, twice a day) | Prednisone (10 mg, po, once a day); HCQ (200 mg, po, twice a day) |
|  | 4 | 824002 | DFPP (2 times) and IA (1 time); Prednisone (20 mg, po, once a day); HCQ (200 mg, po, twice a day) | Prednisone (40 mg, po, once a day); HCQ (200 mg, po, twice a day) | Prednisone (30 mg, po, once a day); HCQ (200 mg, po, twice a day) | Prednisone (20 mg, po, once a day); HCQ (200 mg, po, twice a day) |
|  | 5 | 731545 | DFPP (2 times) and IA (1 time); Prednisone (60 mg, po, once a day); Solu Medrol (0.25g, ivgtt, once a day×6 day + 0.5g, ivgtt, once a day×9 day + 0.16g, ivgtt, once a day×3 day + 0.08g, ivgtt, once a day×3 day);  HCQ (200 mg, twice a day) | Prednisone (45 mg, po, once a day); HCQ (200 mg, po, twice a day) | Prednisone (30 mg, po, once a day); HCQ (200 mg, po, twice a day) | Prednisone (10 mg, po, once a day); HCQ (200 mg, po, twice a day) |
|  | 6 | 858497 | DFPP (2 times) and IA (1 time); Prednisone (50 mg, po, once a day); DXM (10mg, once a day×8 day + 7.5mg once a day×3 day + 5mg once a day×3 day + 2.5mg once a day×3 day); HCQ (200 mg, po, twice a day) | Prednisone (50 mg, po, once a day); HCQ (200 mg, po, twice a day) | Prednisone (35 mg, po, once a day); HCQ (200 mg, po, twice a day) | Prednisone (20 mg, po, once a day); HCQ (200 mg, po, twice a day) |
|  | 7 | 841130 | DFPP (2 times) and IA (1 time); Prednisone (60 mg, po, once a day); DXM (5mg once a day×10 day + 2.5mg once a day×3 day); HCQ (200 mg, po, twice a day) | Prednisone (40 mg, po, once a day); HCQ (200 mg, po, twice a day) | Prednisone (25 mg, po, once a day); HCQ (200 mg, po, twice a day) | Prednisone (10 mg, po, once a day); HCQ (200 mg, po, twice a day) |
| **Non-PE group** | 8 | 819162 | Prednisone (5 mg, po, once a day); HCQ (200 mg, po, twice a day) | Prednisone (5 mg, po, once a day); HCQ (200 mg, po, twice a day) | Prednisone (5 mg, po, once a day); HCQ (200 mg, po, twice a day) | Prednisone (5 mg, po, once a day); HCQ (200 mg, po, twice a day) |
|  | 9 | 834667 | Prednisone (10 mg, po, once a day); DXM (5mg once a day×4 day + 2.5mg once a day×5 day); HCQ (200 mg, po, twice a day) | Prednisone (10 mg, po, once a day); HCQ (200 mg, po, twice a day) | Prednisone (10 mg, po, once a day); HCQ (200 mg, po, twice a day) | Prednisone (10 mg, po, once a day); HCQ (200 mg, po, twice a day) |
|  | 10 | 642352 | Prednisone (60 mg, po, once a day); DXM (5mg once a day×4 day); HCQ (200 mg, po, twice a day) | Prednisone (50 mg, po, once a day); HCQ (200 mg, po, twice a day) | Prednisone (40 mg, po, once a day); HCQ (200 mg, po, twice a day) | Prednisone (30 mg, po, once a day); HCQ (200 mg, po, twice a day) |
|  | 11 | 662264 | Prednisone (30 mg, po, once a day); HCQ (200 mg, po, twice a day) | Prednisone (20 mg, po, once a day); HCQ (200 mg, po, twice a day) | Prednisone (10 mg, po, once a day); HCQ (200 mg, po, twice a day) | Prednisone (15 mg, po, once a day); HCQ (200 mg, po, twice a day) |
|  | 12 | 687221 | Prednisone (30 mg, po, once a day); HCQ (200 mg, po, twice a day) | Prednisone (20 mg, po, once a day); HCQ (200 mg, po, twice a day) | Prednisone (10 mg, po, once a day); HCQ (200 mg, po, twice a day) | Prednisone (45 mg, po, once a day); HCQ (200 mg, po, twice a day) |
|  | 13 | 702006 | Prednisone (45 mg, po, once a day); DXM (10mg, once a day×10 day + 7.5mg once a day×3 day + 5mg once a day×3 day + 2.5mg once a day×3 day); HCQ (200 mg, po, twice a day) | Prednisone (40 mg, po, once a day); HCQ (200 mg, po, twice a day) | Prednisone (30 mg, po, once a day); HCQ (200 mg, po, twice a day) | Prednisone (20 mg, po, once a day); HCQ (200 mg, po, twice a day) |
|  | 14 | 753506 | Prednisone (5 mg, po, once a day); HCQ (200 mg, po, twice a day) | Prednisone (25 mg, po, once a day); HCQ (200 mg, po, twice a day) | Prednisone (20 mg, po, once a day); HCQ (200 mg, po, twice a day) | Prednisone (15 mg, po, once a day); HCQ (200 mg, po, twice a day) |

AD: admission number; HCQ: Hydroxychloroquine Tablets; DFPP: double-filtration plasmapheresis; IA: immunoadsorption; Solu Medrol: Methylprednisolone Sodium Succinate for Injection; DXM: Dexamethasone.


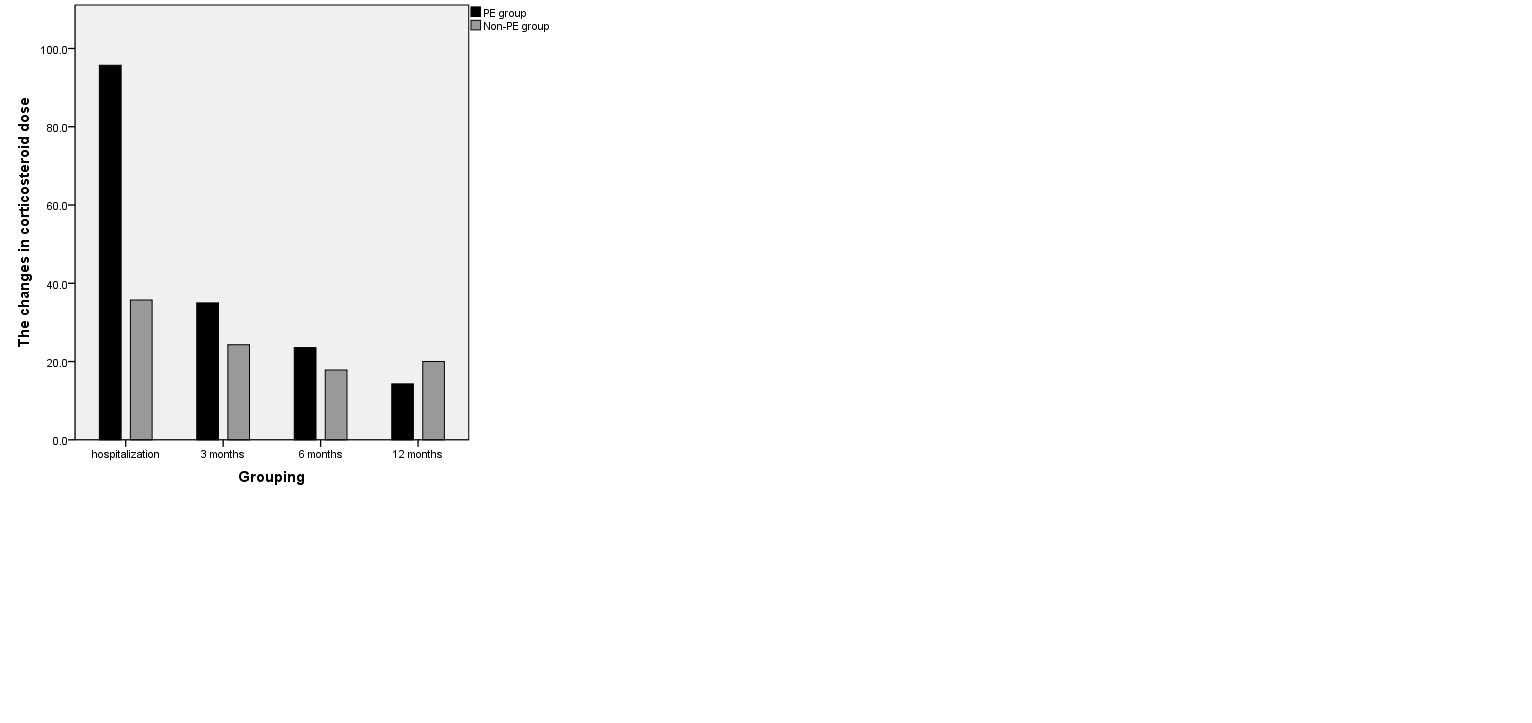


**Figure 1S.** The changes in corticosteroid dose before treatment as well as 3,6 and 12 months after delivery in both groups.
